# Evaluation of Association of *HNF1B* Variants with Diverse Cancers: Collaborative Analysis of Data from 19 Genome-Wide Association Studies

**DOI:** 10.1371/journal.pone.0010858

**Published:** 2010-05-28

**Authors:** Katherine S. Elliott, Eleftheria Zeggini, Mark I. McCarthy, Julius Gudmundsson, Patrick Sulem, Simon N. Stacey, Steinunn Thorlacius, Laufey Amundadottir, Henrik Grönberg, Jianfeng Xu, Valerie Gaborieau, Rosalind A. Eeles, David E. Neal, Jenny L. Donovan, Freddie C. Hamdy, Kenneth Muir, Shih-Jen Hwang, Margaret R. Spitz, Brent Zanke, Luis Carvajal-Carmona, Kevin M. Brown, Australian Melanoma Family Study Investigators, Nicholas K. Hayward, Stuart Macgregor, Ian P. M. Tomlinson, Mathieu Lemire, Christopher I. Amos, Joanne M. Murabito, William B. Isaacs, Douglas F. Easton, Paul Brennan, The PanScan Consortium, Rosa B. Barkardottir, Daniel F. Gudbjartsson, Thorunn Rafnar, David J. Hunter, Stephen J. Chanock, Kari Stefansson, John P. A. Ioannidis

**Affiliations:** 1 Wellcome Trust Centre for Human Genetics, University of Oxford, Oxford, United Kingdom; 2 Applied Statistical Genetics, Wellcome Trust Sanger Institute, University of Cambridge, Cambridge, United Kingdom; 3 Oxford Centre for Diabetes, Endocrinology and Metabolism, University of Oxford, Oxford, United Kingdom; 4 deCODE Genetics, Reykjavik, Iceland; 5 Division of Cancer Epidemiology and Genetics, National Cancer Institute, Bethesda, Maryland, United States of America; 6 Department of Medical Epidemiology and Biostatistics, Karolinska Institutet, Stockholm, Sweden; 7 Center for Cancer Genomics, Wake Forest University School of Medicine, Winston-Salem, North Carolina, United States of America; 8 Center for Human Genomics, Wake Forest University School of Medicine, Winston-Salem, North Carolina, United States of America; 9 Genetic Epidemiology Group, International Agency for Research on Cancer (IARC), Lyon, France; 10 Oncogenetics Team, The Institute of Cancer Research, Sutton, United Kingdom; 11 Department of Oncology, University of Cambridge, Cambridge, United Kingdom; 12 Department of Social Medicine, University of Bristol, Bristol, United Kingdom; 13 Nuffield Department of Surgery, University of Oxford, Oxford, United Kingdom; 14 Health Sciences Research Institute, University of Warwick, Coventry, United Kingdom; 15 Framingham Study, National Heart, Lung, and Blood Institute, Bethesda, Maryland, United States of America; 16 Department of Epidemiology, M. D. Anderson Cancer Center, Houston, Texas, United States of America; 17 Ontario Institute for Cancer Research, MaRS Centre, Toronto, Ontario, Canada; 18 Ottawa Health Research Institute, University of Ottawa, Ottawa, Ontario, Canada; 19 Integrated Cancer Genomics Division, The Translational Genomics Research Institute, Phoenix, Arizona, United States of America; 20 Queensland Institute of Medical Research, Royal Brisbane Hospital, Brisbane, Queensland, Australia; 21 Section of General Internal Medicine, Boston University School of Medicine, Boston, Massachusetts, United States of America; 22 The Brady Urological Institute, Johns Hopkins Medical Institutions, Baltimore, Maryland, United States of America; 23 Cancer Research UK Genetic Epidemiology Unit, University of Cambridge, Cambridge, United Kingdom; 24 Department of Pathology, Landspitali-University Hospital of Iceland, Reykjavik, Iceland; 25 Faculty of Medicine, University of Iceland, Reykjavik, Iceland; 26 Department of Epidemiology, Harvard School of Public Health, Boston, Massachusetts, United States of America; 27 Department of Hygiene and Epidemiology, University of Ioannina School of Medicine and Biomedical Research Institute, Foundation for Research and Technology-Hellas, Ioannina, Greece; 28 Center for Genetic Epidemiology and Modelling, Tufts University School of Medicine, Boston, Massachusetts, United States of America; Innsbruck Medical University, Austria

## Abstract

**Background:**

Genome-wide association studies have found type 2 diabetes-associated variants in the *HNF1B* gene to exhibit reciprocal associations with prostate cancer risk. We aimed to identify whether these variants may have an effect on cancer risk in general versus a specific effect on prostate cancer only.

**Methodology/Principal Findings:**

In a collaborative analysis, we collected data from GWAS of cancer phenotypes for the frequently reported variants of *HNF1B*, rs4430796 and rs7501939, which are in linkage disequilibrium (r^2^ = 0.76, HapMap CEU). Overall, the analysis included 16 datasets on rs4430796 with 19,640 cancer cases and 21,929 controls; and 21 datasets on rs7501939 with 26,923 cases and 49,085 controls. Malignancies other than prostate cancer included colorectal, breast, lung and pancreatic cancers, and melanoma. Meta-analysis showed large between-dataset heterogeneity that was driven by different effects in prostate cancer and other cancers. The per-T2D-risk-allele odds ratios (95% confidence intervals) for rs4430796 were 0.79 (0.76, 0.83)] per G allele for prostate cancer (p<10^−15^ for both); and 1.03 (0.99, 1.07) for all other cancers. Similarly for rs7501939 the per-T2D-risk-allele odds ratios (95% confidence intervals) were 0.80 (0.77, 0.83) per T allele for prostate cancer (p<10^−15^ for both); and 1.00 (0.97, 1.04) for all other cancers. No malignancy other than prostate cancer had a nominally statistically significant association.

**Conclusions/Significance:**

The examined *HNF1B* variants have a highly specific effect on prostate cancer risk with no apparent association with any of the other studied cancer types.

## Introduction

A large number of epidemiological studies have suggested correlations between type 2 diabetes (T2D) and various cancers[1], [2], [3]. Most evidence suggests an inverse correlation between T2D and prostate cancer[4], [5], [6] although not all studies agree on this[7]. Several studies also suggest positive correlations between other cancers and T2D[1], [2], [3]. It is unclear whether these correlations, if true, represent casual relationships and whether they may also reflect some shared genetic background. Recently, with the advent of genome-wide association studies (GWAS), a large number of genetic variants have been identified that confer susceptibility to T2D or specific types of cancer[8]. An interesting observation has been that specific variants in the *HNF1B* gene (formerly *TCF2*) have been demonstrated to be associated both with the risk of prostate cancer[9], [10], [11] and the risk of T2D[9], [12] with the effects being in the opposite direction for these two phenotypes.

*HNF1B* was previously known to be mutated in individuals with maturity-onset diabetes of the young type 5 (MODY 5)[13], but a biological explanation of the impact of the identified common variation on T2D and prostate cancer risk remains elusive. The identified genetic effects are small in magnitude even for prostate cancer and T2D, representing odds ratios [ORs] per allele in the range of 1.2 [9], [11] and 0.9 [9], [12], respectively. Therefore, small effects for other cancer types would not be readily detectable, unless very large studies were performed or data were combined from several studies.

A definitive answer on whether *HNF1B* variants modulate also the risk of other malignancies, or show specificity for prostate cancer, requires large sample sizes. Here we present the results of a large collaborative meta-analysis of *HNF1B*, rs4430796 and rs7501939, which have the most consistent associations with both prostate cancer and T2D. Relevant data were collected on the two variants from GWAS on cancer phenotypes in Caucasian populations in order to examine whether they have an effect on cancer risk in general, on few specific cancers, or only on prostate cancer.

## Results

### Database of contributed information

All the originally contacted investigators of cancer-related GWA studies agreed to participate in this collaborative analysis, with the exception of the investigators of 3 GWA studies [14], [15], [16] (1 on breast cancer, 1 on colorectal cancer and 1 on neuroblastoma), 1 of which had no data on the requested variants, as they had used a Affymetrix platform[15]. Investigators who agreed to participate in the collaborative analysis contributed data on 13 datasets for rs4430796 and 19 datasets for rs7501939 [11], [17], [18], [19], [20], [21], [22], [23], [24], [25], [26], [27], [28], [29], [30], [31], [32], [33]. For 5 datasets, data were available only for the latter polymorphism either because the polymorphism was not available on the platform used or the SNP failed quality control criteria.

The contributing teams and datasets are shown in Table 1 with data on the number of cases and controls for each polymorphism and for each type of cancer. Datasets from the Framingham cohort contained imputed data for both polymorphisms since an Affymetrix platform had been used, rs4430796 data from the M.D. Anderson Cancer Center was imputed since this SNP had not been directly genotyped, and melanoma data from AMFS and Q-MEGA contained counts from pooling experiments, otherwise all other datasets had direct genotyping on individual participants. Detailed demographic and other characteristics of the study populations can be found in the respective primary publications of these GWA studies [14], [15], [16], [17], [18], [20], [21], [22], [23], [24], [25], [26], [27], [28], [29], [30], [31], [32], [34].

**Table 1:** Characteristics of datasets included in the collaborative meta-analysis.

Study Centre	Cancer	Genotyping platform(s)	rs4430796 #cases	rs4430796 #controls	rs7501939 #cases	rs7501939 #controls
*ARCTIC	colorectal[23]	Sequenom homogenous MassExtend (in house)	1,079	1,089	1,075	1,087
*AMFS	melanoma[17], [24]	Illumina 550K (pooled)	490 *^p^*	427 *^p^*	490 *^p^*	427 *^p^*
Cambridge	breast [33]	Perlegen	387	363	387	363
*CGEMS	prostate[11], [25]	Illumina 550K	4,960	5,021	4,869	4,930
*CAPS	prostate[26]	Sequenom (in house)	2,874	1,708	2,865	1,707
*CORGI	colorectal[27]	Illumina 550K	n/a	n/a	900	908
deCODE	breast[28]	Illumina 300K	n/a	n/a	1,815	30,742
deCODE	colorectal[29]	Illumina 300K	n/a	n/a	988	30,742
deCODE	lung[29], [30]	Illumina 300K	n/a	n/a	651	30,742
deCODE	prostate[9], [31], [32]	Illumina 300K	n/a	n/a	1619	30,742
*FHS	breast[34]	Affymetrix 500K and MIPS 50K combined	182*^i^*	852 *^i^*	182 *^i^*	852 *^i^*
*FHS	colorectal[34]	Affymetrix 500K and MIPS 50K combined	108 *^i^*	1,498 *^i^*	108 *^i^*	1,498 *^i^*
*FHS	lung[34]	Affymetrix 500K and MIPS 50K combined	90 *^i^*	1,498 *^i^*	90 *^i^*	1,498 *^i^*
*FHS	prostate[34]	Affymetrix 500K and MIPS 50K combined	190 *^i^*	646 *^i^*	190 *^i^*	646 *^i^*
*IARC	lung[20], [21]	Illumina 300K	641	2,435	1,797	2,378
*JHH	prostate[26]	Sequenom (in house)	1,512	478	1,521	479
*MDACC	lung[22]	Illumina 317K	1,152 *^i^*	1,137 *^i^*	1,152	1,137
*PANSCAN	pancreatic Stage 1[19], [48]	Illumina 550K and 610K	1,754	1,796	1,757	1,796
*PANSCAN	pancreatic Stage 2[19], [48]	Illumina 550K and 610K	1,748	1,818	1,769	1,841
*Q-MEGA	melanoma[24]	Illumina 550K (pooled)	864*^p^*	864 *^p^*	864 *^p^*	864 *^p^*
*UKGPCS	prostate[18]	Illumina 550K	1,609	1,797	1,834	1,867

Unless otherwise indicated all data is from direct genotyping. *ARCTIC (Assessment of Risk for Colorectal Tumors in Canada), AMFS (Australian Melanoma Family Study), CGEMS (Cancer Genetics Markers of Susceptibility), CAPS (Cancer of the Prostate in Sweden), CORGI (Colorectal Tumour Gene Identification), FHS (Framingham Heart Study), IARC (International Agency for Research on Cancer), JHH (Johns Hopkins Hospital), MDACC (M.D. Anderson Cancer Center, Texas), PANSCAN (Pancreatic Cancer Cohort Consortium), Q-MEGA (Queensland study of Melanoma: Environment and Genetic Associations), UKGPCS (UK Genetic Prostate Cancer Study). n/a: no available data; *i*: imputed; *p*: pooled.

Overall, the collaborative analysis included data on rs4430796 for 19,640 cancer cases and 21,929 controls; for prostate cancer there were 11,145 cases and 9,650 controls, while for all other cancers there were 8,495 cases and 12,279 controls. The collected data on rs7501939 included 26,923 cases and 49,085 controls; for prostate cancer there were 12,898 cases and 40,371 controls, while for the other cancers there were 14,025 cases and 43,893 controls. Malignancies other than prostate cancer in these datasets included colorectal, breast, lung and pancreatic cancers, and melanoma (Table 1). deCODE contributed data on 4 different cancers and had a common population control group for all 4 of them. The Framingham Heart Study (FHS) contributed data on 4 different cancers and had a common population control group (subjects ≥65 years at the last contact who are not nuclear family member of the cancer cases) for all 4 studies with the exception of prostate and breast cancer which used male and female only controls respectively. The common control groups for deCODE and FHS are only counted once in the total sample sizes above.

**Figure 1:**
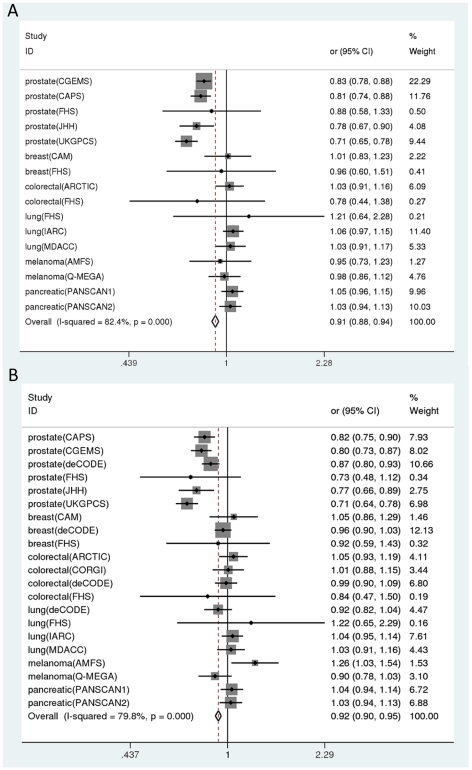
Association of rs4430796 and rs7501939 with diverse cancer types. Panel A shows results for rs4430796 and panel B shows results for rs7501939. Each study is shown by its odds ratio and 95% confidence intervals). Prostate cancer studies appear on the top and other cancer studies follow in alphabetical order. For the abbreviations of the names of the studies see Table 1. The summary diamond at the bottom corresponds to the fixed effects summary. Weight indicates the relative proportion of the total evidence found in each study (the weight is inversely proportional to the variance).

The meta-analysis of all datasets (Table 2, Figure 1) showed a per T2D risk allele association with both rs4430796 (G allele OR 0.91 [95% CI: 0.88, 0.94] p = 3×10^−10^) and rs7501939 (T allele OR 0.91 [95% CI: 0.88, 0.94] p = 5v10^−10^) according to fixed effects calculations, while by random effects calculations there was nominal significance (OR 0.94 [95% CI: 0.88, 1.00], p = 0.033 for rs4430796 and 0.93 [95% CI: 0.86, 1.01], p = 0.07 for rs7501939). The reason for this diversity is that there was very large between-study heterogeneity in the effect sizes (I^2^ of 82% [95% CI: 73-89%] and 80% [95% CI: 70-86%], respectively, for the two polymorphisms; Q-test p-value <0.001 for both polymorphisms), and this makes the fixed effects calculations less reliable. Results were qualitatively similar when we increased the variance in deCODE, FHS, and IARC estimates to account for the overlapping control group (not shown).

**Table 2:** Summary of results for association between rs4430796 and rs7501939 and diverse cancer types.

	rs4430796	rs4430796	rs4430796	rs7501939	rs7501939	rs7501939
Cancer type	Studies (cases, controls)	OR (95% CI)	I^2^ (95% CI)	Studies (cases, controls)	OR (95% CI)	I^2^ (95% CI)
All cancers	16 (19,640, 21,929)*	0.91 (0.88, 0.94)	82 (73, 89)	21 (26,923, 49,085)*	0.92 (0.90, 0.95)	80 (70, 86)
Prostate	5 (11,145, 9,650)	0.79 (0.76, 0.83)	42 (0, 79)	6 (12,898, 40,371)	0.80 (0.77, 0.83)	56 (0, 82)
All Others	11 (8,495, 12,279)*	1.03 (0.99, 1.07)	0 (0, 60)	15 (14,025, 43,893)*	1.00 (0.97, 1.04)	0 (0, 54)
Breast	2 (569, 1,215)	1.00 (0.84, 1.20)	n/a	3 (2,384, 31,957)	0.97 (0.91, 1.04)	0 (0, 90)
Lung	3 (1,883, 5,070)	1.05 (0.98, 1.13)	0 (0, 90)	4 (3,690, 35,755)	1.03 (0.96, 1.10)	0 (0, 85)
Colorectal	2 (1,187, 2,587)	1.01 (0.90, 1.14)	n/a	4 (3,071, 34,235)	1.01 (0.94, 1.08)	0 (0, 85)
Melanoma	2 (1,354, 1,291)	0.98 (0.87, 1.01)	n/a	2 (1,354, 1,291)	1.01 (0.90, 1.13)	n/a
Pancreatic	2 (3,502, 3,614)	1.04 (0.98, 1.11)	n/a	2 (3,526, 3,637)	1.03 (0.97, 1.10)	n/a

OR: odds ratio, CI: confidence interval, n/a: not applicable (heterogeneity I^2^ confidence intervals are not calculated when there are only 2 studies). Odds ratios are based on fixed effects calculations. When point estimates or confidence intervals differ by over 1% in random effects calculations, random effects results are mentioned in the text. * the common control groups of deCODE and FHS are counted only once.

The heterogeneity was largely driven by the diversity in the effect sizes between prostate cancer and all other cancers. A meta-analysis limited to prostate cancer datasets gave consistent associations with both rs4430796 (OR per copy of T2D risk allele (A) 0.79 [95% CI: 0.76, 0.83], p<10^−15^ by fixed effects and 0.79 [95% CI: 0.74, 0.84] p = 10^−13^ by random effects), and rs7501939 (OR per copy of T2D risk allele (T) 0.80 [95% CI: 0.77, 0.83] p<10^−15^ by fixed effects and 0.79 [95% CI: 0.74, 0.85], p = 2×10^−11^ by random effects) (Table 2). There was some residual between-study heterogeneity even within the prostate cancer datasets (I^2^ of 42% [95%CI: 0–79%] and 56% [95% CI: 0–82%], respectively, for the two polymorphisms; Q-test p-value 0.037 and 0.14, respectively), although the heterogeneity pertained only to the exact magnitude of the genetic effects and a nominally statistically significant association was seen in each of the datasets except for the Framingham study where the number of prostate cancer cases was more limited.

Conversely, the results for all other cancers suggested no significant association and results were consistent across studies. The summary OR was 1.03 and 1.00 for the two polymorphisms respectively (p = 0.14 and 0.81 by fixed effects) and the 95% CIs excluded ORs deviating more than 7% from the null (OR = 1.00) for rs4430796 and more than 4% from the null for rs7501939 (Table 2). The Q-test p-value was 0.99 and 0.45 for the two polymorphisms respectively and random effects estimates were thus identical to fixed effects estimates.

There was also no convincing evidence for an association between either of the two polymorphisms and any of the other cancers (besides prostate cancer), when each cancer type was evaluated separately. Point estimates were in the opposite direction (odds ratio 1.03–1.05) for pancreatic and lung cancer, but were not nominally statistically significant (Table 2). The difference between the prostate cancer and other cancers' effect estimates was beyond chance (p<0.05) for both polymorphisms.

## Discussion

The current collaborative analysis documents that both rs4430796 and rs7501939 have robust support for association with prostate cancer, while we did not observe any convincing evidence for an association of any of the other cancers examined with either polymorphism. When data from all other cancers, excluding prostate cancer, were combined the summary effects had 95% CIs that excluded even subtle associations. Apart from prostate cancer, when other datasets for each individual cancer type was combined, the 95% CIs consistently excluded associations with modest effects. This would suggest that the effects mediated by these polymorphisms are specific to T2D and prostate cancer and they do not involve any other cancer types.

The *HNF1B* gene encodes a transcription factor and it was initially identified as a MODY gene[13]. Subsequent studies have suggested that mutations in this gene may also be related to renal disease[35] and chromophobe renal cell carcinoma[36]. No GWAS evaluating kidney cancer were included in our analysis, and no kidney cancer GWAS has been published to-date. The expression profile of the gene shows a tissue-specific pattern. It is essential for embryonic survival and is expressed in the gut, kidney, liver, lung, pancreas, prostate, thymus and genital tract [37], [38]. It could be speculated that the lack of association with some cancers studied here may be due to the low or absent expression of this gene in those tissues (for example breast cancer). We did not have data on liver cancer, thymoma or genital tract cancer, but data on lung, pancreatic, and colorectal cancer showed no association, with point estimates very near to the null.

The two variants that we assessed are not necessarily the functional culprits. GWA studies typically derive markers of phenotypes that are probably linked with the functional genetic variation[39]. However, identifying the functional variants is difficult. Even if they could be identified, it is unlikely that substantially large genetic effects for other cancers would exist, if the tagging variants have so consistently null effects. Another caveat is that we only examined populations of Caucasian descent. This reduces the heterogeneity that could be due to different LD patterns in populations of different ancestry. However, it would be worthwhile to investigate the associations of the *HNF1B* variants for T2D, prostate cancer, and other cancers, also in non-Caucasian populations. Preliminary data suggest that both of the examined variants had consistent associations with T2D in Caucasian, Asian (Hong Kong), and West African ancestry participants[9], while the association of rs4430796 with prostate cancer risk was found to be even stronger in the Japanese than in Caucasian populations[40]. Moreover, it would be useful to dissect associations with specific disease subsets. Even within the analyzed Caucasian-descent populations, we observed some modest between-study heterogeneity in the strength of the association between the *HNF1B* variants and prostate cancer. This may be due to different associations in different sub-phenotypes. For example, some data suggest that the rs4430796 A allele may primarily increase the risk for early-onset (<50 years) prostate cancer rather than later-onset disease[41].

In conclusion, while the two examined *HNF1B* variants conclusively have pleiotropic effects on both T2D and prostate cancer, the pleiotropy apparently does not extend to other cancer types. Genetic effects may offer a way to dissect comorbidity between specific cancers and metabolic phenotypes. Besides *HNF1B*, other gene loci have started appearing where variants are identified that modulate susceptibility to both T2D and some malignancy, e.g. prostate cancer for the *JAZF1* locus gene [11], [42] and melanoma for the *CDKN2A* locus [43], although different, unlinked variants are implicated in the susceptibility to the malignancy and T2D, respectively. The elucidation of correlated pleiotropic effects on diverse phenotypes will require very large studies, given the generally subtle effects involved. Collaborative efforts between multiple teams, as in the current study, may offer a suitable approach to answer such questions.

## Methods

### Eligible GWA investigations and data

We used the NHGRI catalogue of published GWA studies[44], a comprehensive database of GWA investigations to identify GWA studies on cancer phenotypes published as of May 20, 2008. We also performed additional PubMed searches to identify whether any additional GWA studies on cancer phenotypes had been published until then. We focused on solid cancers, excluding hematologic malignancies. Given that these GWAS did not include any studies on pancreatic cancer (of special interest, given the association with T2D), we also identified GWAS on pancreatic cancer that had not been published by that time, so as to ensure their inclusion.

We communicated with the corresponding and principal investigators of all of these studies to request their participation in the collaborative meta-analysis. The investigators of these studies were asked to contribute relevant data on genotype frequencies in cancer cases and non-cancer controls for the *HNF1B* variants, rs4430796 and rs7501939. The risk alleles for prostate cancer are A and C for rs4430796 and rs7501939 respectively. The risk alleles for T2D are G and T for rs4430796 and rs7501939 respectively. The two SNPs have modestly high LD in Caucasians, but low LD in Africans (r^2^ = 0.77 and 0.22 in CEU and YRI, respectively). Investigators were requested to provide all GWA data that they had obtained for evaluation of any cancer phenotype, including any additional unpublished datasets. Additional genotyping for the two specific variants was encouraged, when a GWA platform had been used that did not directly genotype these polymorphisms (e.g. Affymetrix or Perlegen rather than Illumina). When a study had data on more than one cancer type, data were requested to be provided separately for each cancer type. Investigators were asked to provide also information and clarifications about the design of their studies, and to ensure that population stratification and cryptic relatedness had been appropriately addressed and appropriate quality controls were available for the genotyping. All GWAS investigations that contributed data on these SNPs used stringent QC standards (as described in detail in their original publications) and the two SNPs fulfilled these standards. Approval from local institutional review boards and steering committees was obtained, as deemed necessary for each study by its investigators. The contributed data were checked for completeness and with logical queries and any missing or unclear information was clarified through communication with the contributing investigators.

### Meta-analysis

For each SNP, we performed meta-analyses including the data from all eligible cancer studies (regardless of the specific cancer phenotype addressed) and also subgroup meta-analyses, with each subgroup limited to studies addressing a specific cancer phenotype. A separate analysis compared the results of the association for prostate cancer versus the association for all other cancers combined.

All analyses followed the per allele (log-additive) model of inheritance with effect sizes expressed in the odds ratio (OR) scale using both fixed and random effects models[45]. Heterogeneity testing used the Q statistic (considered statistically significant at p<0.10), and the I^2^ metric[46] and its 95% CIs [47]. Analyses excluding data from studies with pooled genotyping gave similar results (not shown).

Based on the accumulated total sample size and given the minor allele frequencies of these two polymorphisms in HapMap CEU (47% for rs4430796 A allele and 47% for rs7501939 T allele), the meta-analysis had 95% or higher power to detect an association of OR = 1.10 at alpha = 0.05 with each of the two polymorphisms for overall cancer risk, prostate cancer risk, or other cancer risk. Reported p-values are two-tailed. Analyses were performed in STATA 10.0 (College Station, Texas).
